# Short-term impact of BREF-ED, an early, single-family psychoeducational programme for caregivers of individuals with eating disorders: A retrospective pilot study

**DOI:** 10.1192/j.eurpsy.2025.10089

**Published:** 2025-08-22

**Authors:** Elisabetta Scanferla, Juliette de Salle, Johana Monthuy-Blanc, Rossella Letizia Mancusi, Louis-Ferdinand Lespine, Romain Rey, Philip Gorwood

**Affiliations:** 1 CRPMS (EP), Université Paris Cité, Paris, France; 2 GHU Paris Psychiatrie et Neurosciences, CMME, Hôpital Sainte-Anne, Paris, France; 3 Unité de Recherche Loricorps, Centre de Recherche de l’Institut Universitaire en Santé Mentale de Montréal (CR-IUSMM), Montreal, QC, Canada; 4 Département des Sciences de l’éducation, Université du Québec à Trois-Rivières, Trois-Rivières, QC, Canada; 5 DRCI, GHU Paris Psychiatrie et Neurosciences, Paris, France; 6 Centre Lyonnais des Aidants en Psychiatrie (CLAP), Le Vinatier-Psychiatrie Universitaire Lyon Métropole, Bron, France; 7 Fondation FondaMental, Créteil, France; 8 INSERM, U1028; CNRS, UMR5292, Université Lyon 1; Centre de Recherche en Neurosciences de Lyon, équipe PsyR2, Lyon, France; 9 Institut de Psychiatrie et Neurosciences de Paris (IPNP), Université Paris Cité, INSERM U1266, Paris, France

**Keywords:** burden, caregivers, depression, eating disorders, family psychoeducation

## Abstract

**Background:**

There is an urgent need to improve early accessibility to psychoeducational interventions for informal caregivers of individuals with eating disorders (EDs). We adapted the BREF programme, a short, single-family, psycho-educational intervention originally developed for caregivers in severe mental disorders, to EDs (BREF-ED) and assessed at diagnosis announcement. We hypothesised that it has a good acceptability and effectiveness in reducing short-term caregivers’ self-reported levels of burden and depressive symptoms.

**Methods:**

Data of caregivers who participated in the BREF-ED programme were analysed. Adherence, satisfaction, and perceived usefulness were evaluated. Changes in self-reported burden and depression symptoms were measured pre-, post-, and 3 months after the intervention using the Zarit Burden Interview (ZBI) and Center for Epidemiological Studies – Depression scale (CES-D).

**Results:**

Of the 53 caregivers included in the study, 52 participants completed the BREF-ED programme. As compared to baseline, ZBI scores showed a significant reduction after the intervention (Cohen’s *d* = 0.61, *p* < 0.001), and at the 3-month assessment (Cohen’s *d* = 0.62, *p* < 0.001). The CES-D scores also significantly decreased by the end of the third session (Cohen’s *d* = 0.83, *p* < 0.001) and at the 3-month follow-up (Cohen’s *d* = 0.77, *p* < 0.001). Satisfaction scores were high, with 90.1% of participants reporting being “very satisfied” and 9.9% “satisfied.”

**Conclusions:**

Preliminary findings demonstrated high adherence rates, caregiver satisfaction, and a positive impact on burden and related depressive symptoms immediately after the programme and at short-term follow-up. This time- and resource-efficient programme has the potential for easy dissemination.

## Introduction

Eating disorders (EDs) are serious and complex mental health conditions that typically emerge in adolescence or early adulthood [1] and may become chronic if not adequately addressed [2–4]. The role of informal caregivers in supporting individuals with an ED is crucial [5–7]. Caregivers not only help guide their loved ones towards diagnosis and treatment but also provide essential emotional and practical support throughout the care process, significantly influencing treatment outcomes [7–10]. Nevertheless, the caring responsibility exerts a long-term negative effect on the mental and physical health of caregivers [11, 7, 12], potentially diminishing their capacity to continue providing effective support [11–13]. Caregivers frequently report feeling overwhelmed by the loved one’s denial of symptoms, dysregulated behaviours, and ambivalence towards care interventions [14, 15]. They often feel scared about their loved one’s future [16] and face various challenges, including difficulties finding, navigating, and coordinating treatment and services [17–19], health and financial issues [20], and disruptions to their familial and social relationships [21, 22]. Notably, caregivers’ emotional responses to the symptoms of their relative may inadvertently perpetuate the disorder and undermine the relationship [23, 2, 24–26]. The cumulative effects underline the necessity of providing help to caregivers to fulfil their role while also safeguarding their own well-being [27, 28].

Various interventions have been developed to support caregivers and prevent burden development. Among them are family psychoeducation (FPE) [29–31] and family-based treatments, which have been historically recommended for adolescents with EDs [32–34] and are now suggested as a treatment option for young adults [33, 35–37]. Psychoeducational interventions, even in brief formats, have proven effective in equipping caregivers with the knowledge and skills necessary to support their relatives sustainably, thereby preventing or reducing their distress and the burden associated with their caregiving role [38–40]. Additionally, FPE improves family functioning and positively impacts care outcomes for the loved one [38, 41, 42, 21], including relapse prevention and treatment adherence [32, 43–45]. Given the beneficial impacts of FPE, international recommendations advocate for early and systematic interventions to support caregivers of individuals with serious mental disorders, including EDs [21, 34–36, 46].

Despite the well-documented benefits of FPE, its availability remains limited in many countries [14]. In France, among 4.5 million caregivers of individuals living with a severe mental disorder (SMD), only a very small percentage (3%) have benefited from FPE [46, 47]. On average, the time before caregivers receive this support is around 10 years after the illness onset. In the context of EDs, these barriers are intensified by the challenging access to specialist care [48] and the long waiting lists for group interventions, including FPE [49]. In France, the absence of systematic FPE provision may be due to the characteristics of the available FPE offer. Indeed, many existing FPE programmes are often not suitable for early and systematic delivery due to their long-lasting format [42, 50] and high resource allocation requirements [38, 51–53]. As a result, many are deprived of the assistance they need and lack early connection with the available resources, especially those provided by family associations (i.e., peer-led organisations supporting family members, caregivers, and their loved ones). This gap in care has been largely highlighted in the literature [22, 43, 54, 55]. To meet this need, a short psychoeducational programme called “BREF” was recently developed in France, in collaboration with the French family association “UNAFAM” (Union nationale de familles et amis de personnes malades et/ou handicapées psychiques) [56]. BREF is a single-family, in-person, FPE programme designed for caregivers of individuals living with SMDs to provide systematic support and basic knowledge about the disorder of the loved one, including symptoms, care options, and caregiving challenges. BREF is complementary to long-format FPE programmes; it aims to increase the flow of caregivers accessing such programmes while reducing delays. It also facilitates early connection of caregivers with peer support groups and family associations for ongoing assistance. The programme is designed and requires minimal levels of professional resources, enhancing the ability of inadequately resourced healthcare services to offer the required support. A preliminary study involving 303 caregivers, primarily parents and spouses of individuals with SMDs, such as schizophrenia, bipolar disorder, depression, and borderline personality disorder, found that the programme significantly reduced participants’ burden and depressive symptoms, with high levels of satisfaction reported [57]. However, empirical evidence is still needed to determine whether this brief intervention can meet the early support needs of caregivers of people with EDs. To bridge this gap, the programme was adapted to suit the context of EDs, resulting in the “BREF-ED” variant. A pilot study was deemed necessary to test the programme’s relevance before a more comprehensive assessment. We, therefore, conducted a pilot study to evaluate the relevance of the BREF-ED programme for caregivers in the context of their loved one’s diagnosis announcement, focusing on its acceptability and potential impact on reducing burden and related self-reported depressive symptoms.

## Materials and methods

### Participants and recruitment

The participants in the programme were caregivers of patients of the CMME, a centre specialised in the diagnosis and treatment of EDs in a French university hospital group (GHU Paris Psychiatrie et Neurosciences). The caregivers were informed about the BREF-ED programme through information material widely available in this centre. Enrolment in the programme was voluntary, with caregivers choosing to participate on their own initiative. All caregivers who consecutively participated in the BREF-ED programme between January 2023 and June 2024 were assessed for eligibility. To be eligible for inclusion in the study, participants had to be: (1) a relative or friend of an individual diagnosed with EDs based on the Diagnostic and Statistical Manual, Fifth Edition [58], as assessed by trained clinicians, and (2) aged 18 years or older. No exclusion criteria were applied.

### Intervention

Similar to the original BREF programme, BREF-ED is a three-session, in-person, single-family programme using specifically designed illustrated cards to explore various themes. Notably, it is conducted without the relative living with an ED. Typically, the first three sessions occurred within a timeframe of 6–8 weeks. During the first session of the programme, caregivers share their experiences and challenges. The second session focuses on the loved one’s symptoms, treatment options, and care organisation. The third session addresses the caregivers’ own needs, providing strategies for their well-being, and motivating them in connecting with relevant support resources (long-format FPE programmes and family associations). Three months after the final session, the programme facilitators follow up to review progress and encourage ongoing engagement with support networks.

We adapted the illustrated cards developed for the BREF programme to address the needs of caregivers [16]. Using a participatory approach, a team of ED specialists, along with individuals living with EDs and their relatives, identified 28 key challenges faced by caregivers. These topics were organised into two decks. Deck 1 covered challenges caregivers face directly related to their loved one’s symptoms, attitudes, and care. Deck 2 focused on issues affecting the caregivers’ own lives and well-being. These cards were tested with the caregivers participating in the programme. The frequency of each card’s selection was monitored until data saturation was reached after the 12th cycle of the BREF-ED programme. For Deck 1, the most frequently chosen topics, in descending order, were as follows: the relative’s fear and anxiety; false-self functioning; ambivalence towards care; body dysmorphia; food restrictions; hyperactivity; denial of symptoms; perfectionism; and consequences of the nutritional deficiencies. For Deck 2, the most commonly selected topics included, in descending order, the following: social withdrawal; difficulty navigating the care system; fatigue/helplessness; disruption to family life; guilt; fear for their loved one’s future; the relative’s fragility; lack of understanding of the care; and care-related costs. The most frequently selected cards from each deck were formally included in the BREF-ED programme. Each session of the BREF-ED was co-facilitated by a senior psychologist specialised in EDs and a certified peer support worker, who received 8 h of structured training in the programme [59].

### Measurements and procedure

**Figure 1.**
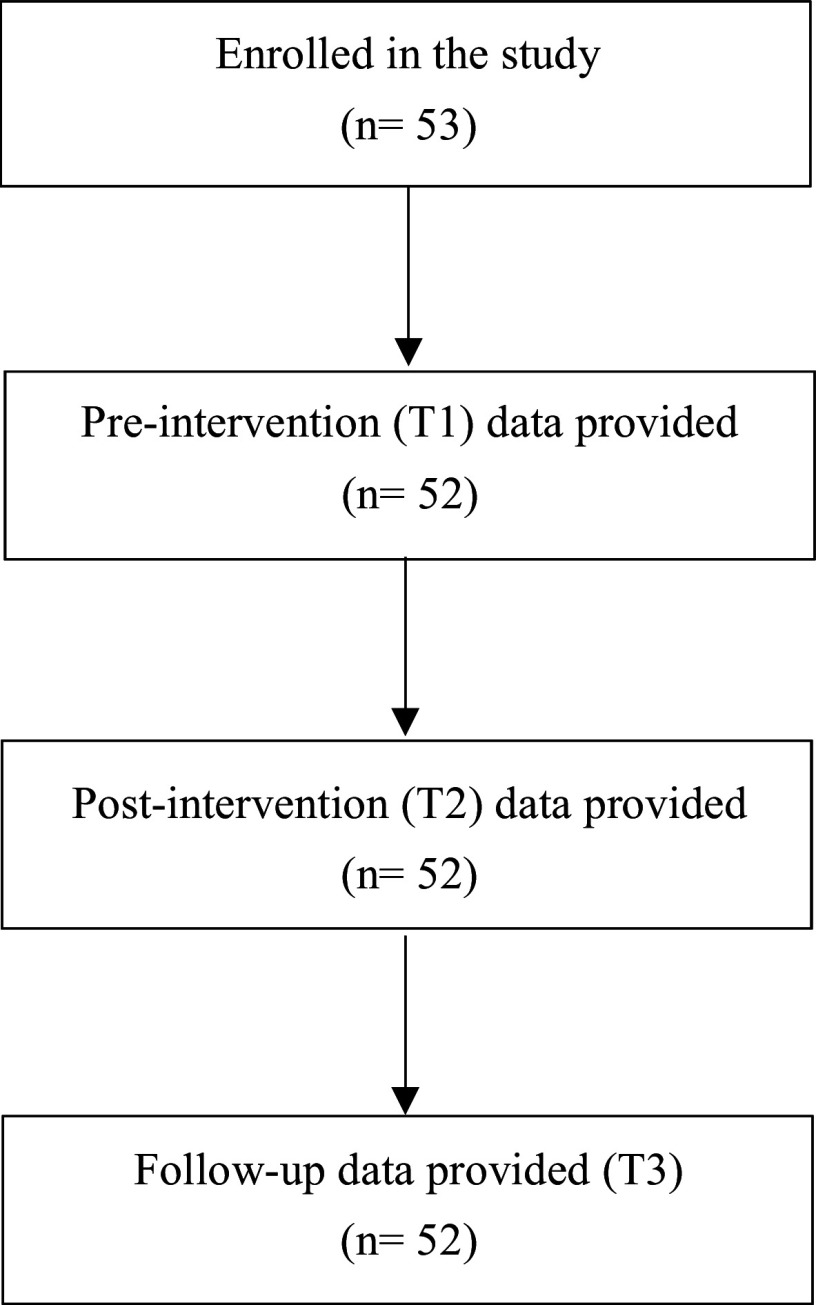
Flowchart of participants’ selection.

Demographic information of the participants enrolled in the programme (age and gender) and their relationship to the relative were collected at baseline. Depression level and caregiving burden were measured at three time points: before the first session (pre-questionnaire; T1), immediately after the third session (post-questionnaire; T2), and 3 months following the intervention (follow-up questionnaire; T3). Participants’ satisfaction and perceived usefulness of the programme were assessed at the follow-up call. The flowchart of participants through each phase of the study is presented in Figure 1.

#### Burden

Caregiving emotional and material burden was measured using the 22-item version of the Zarit Burden Interview (ZBI) [60, 61], a self-report tool in which participants indicate how often various statements such as “you feel that your relative asks for more help than he/she needs” or “you feel embarrassed about your relative’s behaviour” applied to them. Each item is rated on a 5-point Likert scale that ranges from 0 (“*never*”) to 4 (“*nearly always*”).

#### Depression

Depressive symptoms were assessed using the 20-item Center of Epidemiological Studies – Depression (CES-D) scale [62, 63], a self-report questionnaire widely used for evaluating potential signs of depression. Participants indicate how often they experienced feelings like loneliness or sadness in the past week. Responses are given on a 4-point Likert scale ranging from 0 (“*rarely*” or “*none of the time*”) to 3 (“*most of the time*”), with the total score ranging from 0 to 60.

#### Satisfaction and perceived usefulness

Participants rated their satisfaction on a 4-point Likert scale with the options: “*dissatisfied*,” “*neither satisfied nor dissatisfied*,” “*satisfied*,” and “*very satisfied.*” The degree of perceived usefulness of the programme is evaluated on a scale of 1 to 10, with 1 indicating an absence of usefulness and 10 signifying that the programme is “*extremely useful.*”

### Ethical consideration

The study protocol was reviewed and approved by the GHU Paris Psychiatrie et neurosciences Ethics Committee, in accordance with the current ethical guidelines for medical and health research in France (Approved ID: 2024-CER-A-038). All participants expressed written informed consent for the use of their health data in the present study after they were briefed during an in-person meeting.

### Statistical analyses

This study used a single-arm feasibility trial design. The dataset comprised the responses of caregivers who were enrolled in the BREF-ED programme in consecutive order between February 2023 and June 2024 and who had completed the programme by the end of June 2024. No data were missing. The statistical analysis was conducted using the statistical software SPSS, version 26. Descriptive statistical tests were employed to summarise the demographic characteristics of the patients and caregivers. Changes in CES-D and ZBI scores across time (baseline, post-treatment, and follow-up) were analysed using repeated-measures analysis of variance (ANOVA), with effect sizes reported as eta squared (*η*^2^). To explore specific time-point differences, pairwise *post-hoc* comparisons were conducted with Bonferroni correction, effect sizes reported as Cohen’s *d.* The Cochran’s *Q* test was used to assess overall changes in the proportions of individuals classified as having a depressed status (i.e., CES-D score ≥ 16) [64] and those with moderate-to-severe burden (ZBI score ≥ 41) over time, followed by pairwise McNemar tests with Bonferroni correction to adjust for multiple comparisons.

## Results

### Adherence and characteristics of the sample

Fifty-three caregivers voluntarily enrolled in the BREF-ED programme and were included in the study. Among them, 52 participants, related to 29 patients, completed the programme, including the follow-up call conducted 3 months after the final session. Their mean age was 50.6 years (standard deviation [SD] = 12.9), and they were predominantly female (63.5%). The majority of them were the patient’s parents or in-laws (*n* = 41, 78.8%). In eight cases (15.4%), they were siblings, and for only three patients (5.8%), the caregiver was the spouse. The mean age of the family member they care for was 22.3 years (SD = 4.5). The types of EDs their loved ones experienced were: anorexia nervosa (58.6%) and bulimia nervosa (41.4%). Of the included caregivers, 44.2% benefited from the BREF-ED programme <1 year after the first contact of their relative with mental health services. Among the 29 BREF-ED programmes, 18 (62.1%) welcomed between 2 and 4 caregivers (all members of a programme caring for the same relative), while 11 (37.9%) had only one caregiver. The demographic characteristics of the programme participants are presented in Table 1.

**Table 1.** Demographic characteristics of the programme participants (*n* = 52)

Age, mean (SD)	50.6 (12.9)
Gender, *n* (%)	
Female	33 (63.5)
Male	19 (36.5)
Relationship to the relative, *n* (%)	
Parent/parent-in-law	41 (78.8)
Sibling	8 (15.4)
Husband/partner	3 (5.8)
Age of the relative living with an ED, mean (SD)	22.3 (4.5)
Time elapsed between the first contact of the relative with the specialised ED services and the provision of the BREF-ED programme, number of caregivers (%)	
<6 months	19 (36.5)
6–12 months	4 (7.7)
>12 months	29 (55.8)

Abbreviations: ED, eating disorder; *N*, number; SD, standard deviation.

### Changes in burden and depression scores

#### ZBI scores

**Figure 2.**
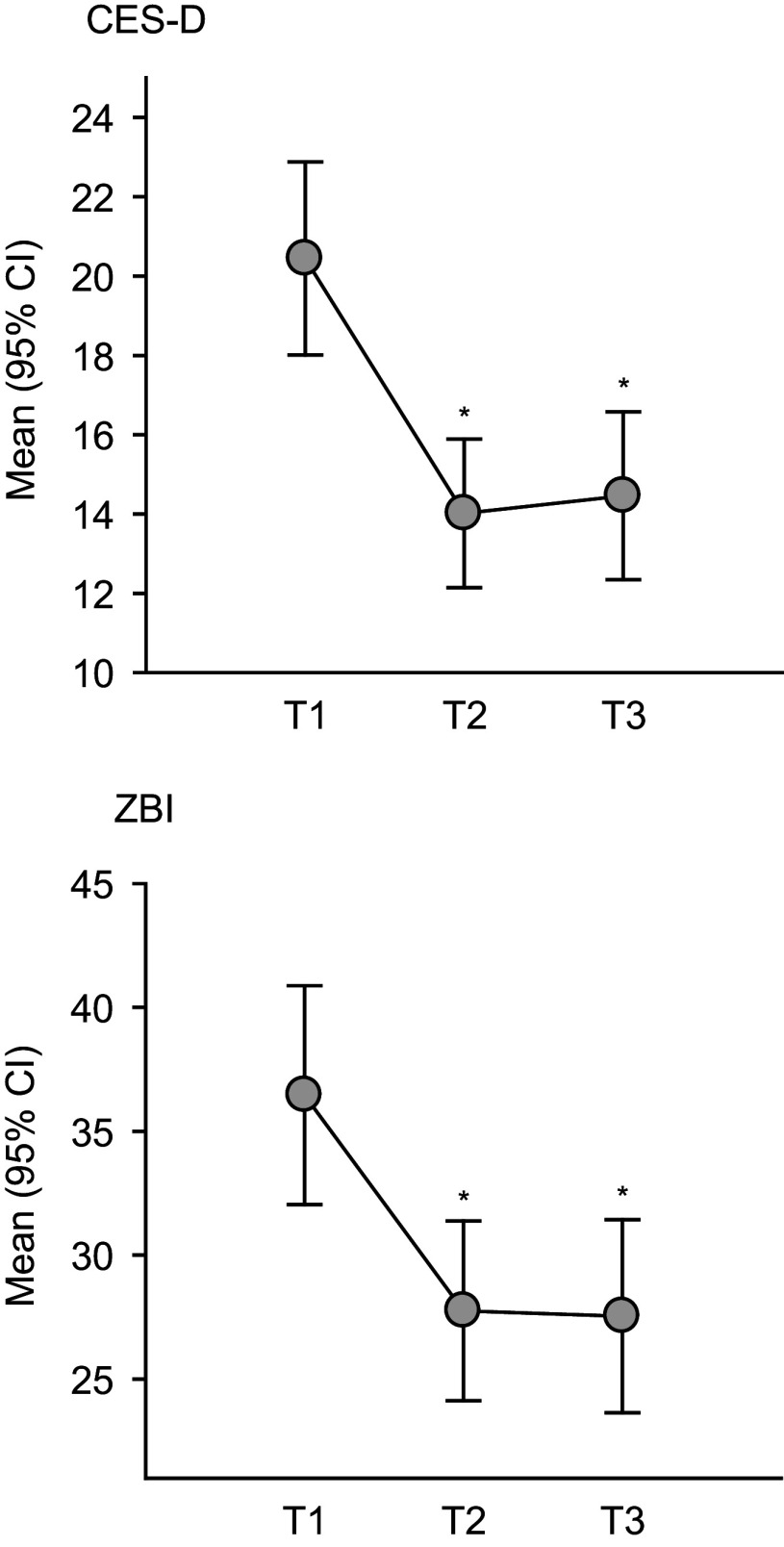
Changes in participants’ mean self-assessed depression (CES-D) and burden (ZBI) before intervention (T1), post intervention (T2), and at follow-up (T3).

**Figure 3.**
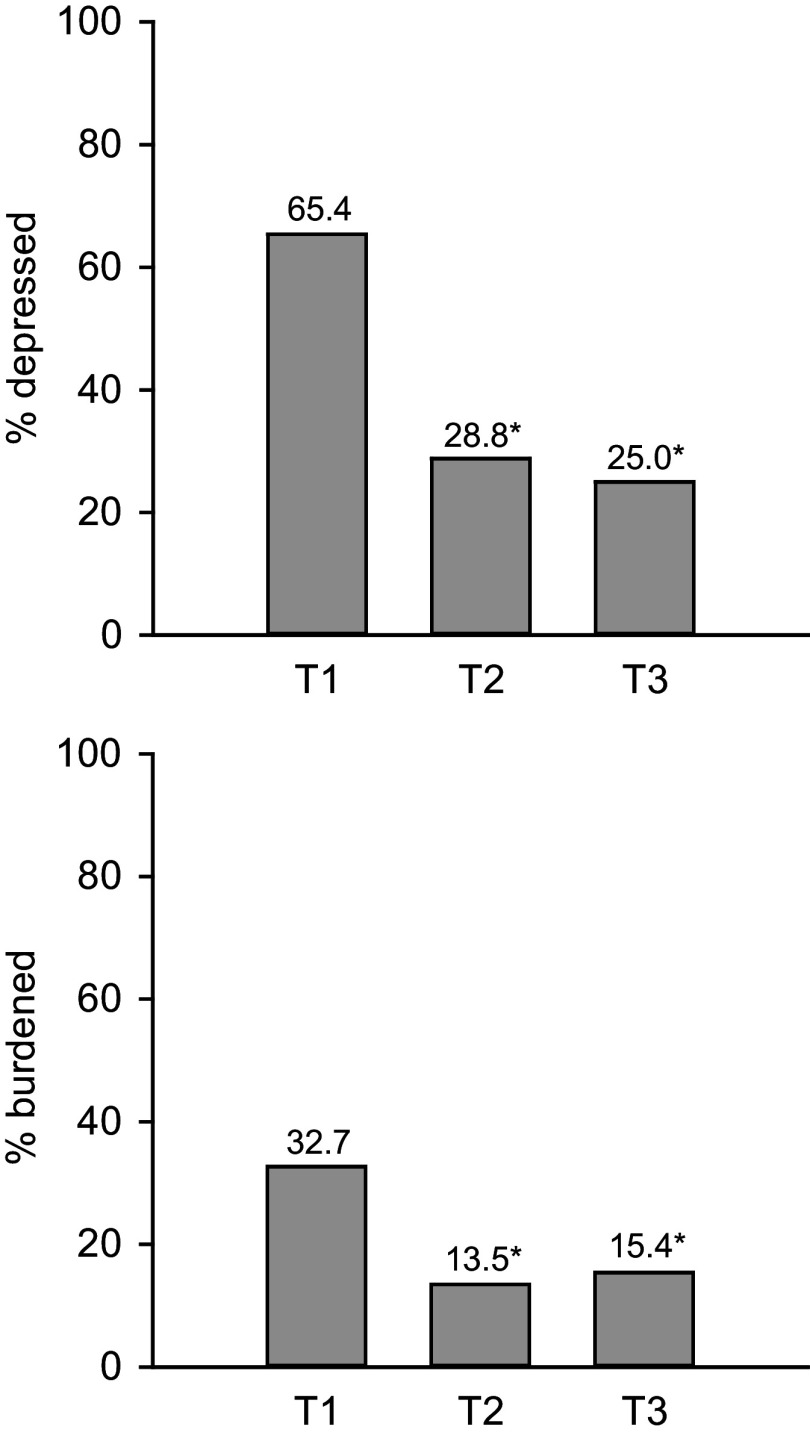
Changes in proportions of depressed (CES-D ≥ 16) and burdened (ZBI ≥ 41) participants before intervention (T1), post intervention (T2), and at follow-up (T3).

The results showed a significant and large effect of time on ZBI scores (F_(2, 102)_ = 19.740, *p* < 0.001, *η*^2^ = 0.279). Post-hoc pairwise comparisons revealed a medium-to-large reduction in ZBI scores from baseline to post-treatment (mean difference = − 8.7, SD = 12.0, adjusted *p* < 0.001, *d* = 0.61) and baseline to follow-up (mean difference = −8.9, SD = 12.7, adjusted *p* < 0.001, *d* = 0.62), but no significant difference between post-treatment and follow-up (mean difference = −0.2, SD = 10.3, adjusted *p* = 1, *d* = 0.02). Results are presented in Table 2 and Figure 2. At baseline, the proportion of caregivers with moderate-to-severe burden (ZBI scores ≥ 41) was 32.7%. This proportion decreased to 13.5% at post-treatment and to 15.4% at follow-up. The Cochran’s *Q* test indicated a significant difference in burden proportions across the time points (*Q*_(3)_ = 139.81, *p* < 0.001). There was a significant reduction in the proportion of burdened caregivers between baseline and both post-treatment (McNemar’s *χ*^2^_(1)_ = 6.750, adjusted *p* = 0.028) and follow-up (McNemar’s *χ*^2^_(1)_ = 5.818, adjusted *p* = 0.047). However, no significant difference between post-treatment and follow-up was observed (McNemar’s *χ*^2^_(1)_ = 0, adjusted *p* = 1) (Figure 3). Contingency tables are reported in the Supplementary Materials.

**Table 2.** Changes in participants’ depressive scores (CES-D) and level of burden (ZBI) before intervention (T1), post intervention (T2), and at follow-up (T3)

	T1	T2	T3	Statistical analyses			
	*M* (SD)	*M* (SD)	*M* (SD)	Time effect^a^	T0 vs. T1	T0 vs. T2	T1 vs. T2
CES-D	20.4 (8.7)	14.0 (6.7)	14.5 (7.6)	F_(1.64, 83.74)_ = 18.729	−6.4 (8.0)	−6.0 (10.2)	+0.4 (6.9)
				**adj-*p* < 0.001**	**adj-*p* < 0.001**	**adj-*p* < 0.001**	adj-*p* = 1
				*η*^2^ = 0.269	*d* = 0.83	*d* = 0.77	*d* = 0.06
ZBI	36.5 (15.9)	27.8 (13.0)	27.5 (14.0)	F_(2, 102)_ = 19.740	−8.7 (12.0)	−8.9 (12.7)	−0.2 (10.3)
				**adj-*p* < 0.001**	**adj-*p* < 0.001**	**adj-*p* < 0.001**	adj-*p* = 1
				*η*^2^ = 0.279	*d* = 0.61	*d* = 0.62	*d* = 0.02

Abbreviations: CES-D, Center of Epidemiological Studies – Depression; *M*, mean; *η*^2^*p*, partial eta squared; SD, standard deviation; T1, before intervention; T2, post intervention; T3, follow-up (T3); ZBI, Zarit Burden Interview.

a Bonferroni-adjusted *p*-values. Effect sizes reported as Cohen’s *d.*

#### CES-D scores

A repeated-measures ANOVA was conducted to examine the effect of time (baseline, post-treatment, and follow-up) on CES-D scores. Mauchly’s test indicated a violation of the sphericity assumption, *χ*^2^_(2)_ = 12.298, *p* = 0.002. Therefore, a Greenhouse–Geisser correction was applied (*ϵ* = 0.821). The results showed a significant and large effect of time on CES-D scores (F_(1.64, 83.74)_ = 18.729, *p* < 0.001, *η*^2^ = 0.269). Post-hoc pairwise comparisons revealed a large reduction in CES-D scores from baseline to post-treatment (mean difference = −6.4, SD = 8.0, adjusted *p* < 0.001, *d* = 0.83) and baseline to follow-up (mean difference = −6.0, SD = 10.2, adjusted *p* < 0.001, *d* = 0.77), but no significant difference between post-treatment and follow-up (mean difference = 0.4, SD = 6.9, adjusted *p* = 1, *d* = 0.06). Results are presented in Table 2 and Figure 2.

At baseline, 65.4% of participants were experiencing a depressive episode that required professional intervention. This proportion decreased to 28.8% at post-intervention and to 25% at follow-up. The Cochran’s *Q* test indicated a significant difference in depression proportions across the time points (*Q*_(3)_ = 125.42, *p* < 0.001). There was a significant reduction in the proportion of caregivers experiencing depressive symptoms between baseline and both post-treatment (McNemar’s *χ*^2^_(1)_ = 12.960, adjusted *p* < 0.001) and follow-up (McNemar’s *χ*^2^_(1)_ = 12.903, adjusted *p* < 0.001). However, no significant difference between post-treatment and follow-up was observed (McNemar’s *χ*^2^_(1)_ = 0.083, adjusted *p* = 1) (Figure 3). Contingency tables are reported in the Supplementary Materials.

### Satisfaction and perceived usefulness of the intervention

Participants’ satisfaction and perceived usefulness of the programme were assessed at follow-up. Adherence rates were excellent, with 98.1% participants completing the three programme sessions and the follow-up call 3 months after the final session. All completers reported being “satisfied” (9.9%) or “very satisfied” (90.1%) with the BREF-ED programme. The mean level of perceived usefulness was 9.2 points (±1.5) on a 10-point scale, indicating that participants perceived the programme as highly useful. None of the participants considered the programme to be useless.

## Discussion

This is the first study to investigate a short psychoeducational programme (BREF-ED) designed for early and systematic delivery to caregivers of individuals with EDs. A retrospective analysis was conducted on data from participants enrolled in this single-family support intervention following the announcement of their loved one’s diagnosis. Adherence rates were excellent, with 98.1% participants completing the three programme sessions and the follow-up call 3 months after the final session. All completers reported being either “satisfied” or “very satisfied” and acknowledged the valuable support it provided. A significant reduction in caregivers’ burden and self-reported depressive symptoms was observed following the programme, with a significant decrease in the proportion of caregivers reporting moderate-to-severe burden or experiencing clinical depressive symptoms.

The study’s results support the acceptability and feasibility in an acute care context of a single-family FPE programme intended to be provided promptly and widely. Notably, the single-family format enables a personalised intervention, designed to meet the individual needs of each family. Consistently, feedback analysis revealed that caregivers particularly valued the opportunity for open discussions about their unique caregiving challenges, the provision of targeted knowledge (e.g., information about their loved one’s specific symptoms, communication strategies, etc.) [65], the blend of “scientific” and experiential knowledge [66, 67], and the high level of reassurance and discretion favoured by the single-family specific format. Additionally, the BREF-ED programme settings, particularly its short format, facilitate rapid access, which is especially beneficial when prompt and need-driven support is crucial [36, 68]. It is worth noting that, as a short intervention, the BREF programme is very different and cannot replace a longer FPE programme that has demonstrated its effectiveness in long-term improvement of depression and burden. In accordance with the pyramid of family care model [69], BREF should rather be regarded as an initial intervention, complementary to longer family support interventions, which aims to motivate caregivers to engage in longer FPE programmes. Indeed, the single-family format offers a flexible and agile approach, broadening the range of early support options available to caregivers within healthcare services [70].

The findings of the present study are consistent with those of Rey et al. [56, 57, 59] on the original BREF programme for caregivers of individuals with SMD, which reported a positive impact on burden and depressive symptoms experienced by relatives. These results also align with existing literature, which highlights that FPE programmes may exert a positive impact on caregivers’ depressive symptoms, possibly via reducing their burden [48].

Furthermore, the results of this study also showed that a significant proportion of caregivers in psychiatry face high levels of burden and depressive symptoms, which is confirmed by previous research on those providing care to individuals with EDs [7, 11, 15, 21, 25]. Notably, in the current study, 65.4% of participants were experiencing clinically significant depressive symptoms at baseline. This is significantly higher than the proportions (13–30%) of caregivers scoring above the clinical threshold for depression reported in earlier studies [11, 71–75]. This might be because 44.2% of the caregivers included in this study benefited from the BREF-ED programme during the first year following their loved one’s diagnosis of an ED, often in a severe form. This timing may partly explain the high proportion of participants reporting burden and clinical depressive symptoms, as they faced the immediate emotional impact of the diagnosis and the pressure of seeking appropriate care. Altogether, these results highlight the high vulnerability of caregivers of individuals with EDs and the necessity of supporting them systematically and at the earliest possible stage of their caregiving role [21, 34, 38, 76]. Notably, the present study did not capture long-term outcomes, which could potentially be more negative for caregivers of patients with a more chronic and severe illness course. For these caregivers, we hypothesise that BREF-ED may enable an earlier and more systematic referral to family associations and long-format FPE programmes, thereby providing them with sustainable support. However, further research is needed to determine whether the BREF-ED programme serves as a pivotal link in shaping the caregivers’ journey with family psychoeducation.

Overall, the findings of the present study support the acceptability and feasibility of the BREF-ED programme, as well as its potential benefits for caregivers of individuals experiencing severe EDs in an acute context. This programme is low time- and resource-consuming, which are critical characteristics for systematic interventions.

This study has several limitations. First, its retrospective design, small sample size, lack of randomisation, and a control group restrict the generalisability of the findings. However, they provide sufficient evidence for a further investigation through a multi-site randomised controlled trial. Including a waitlist control group would help determine whether external factors or the natural progression of the caregiving experience influenced changes in caregiver burden and depressive symptoms. This research would provide more robust evidence of the BREF-ED programme’s impact and support the benefits of its broader inclusion in healthcare services. Second, the included participants were primarily caring for individuals with anorexia nervosa and bulimia nervosa. This reflects the patient population treated at the specialised centre where they were recruited. Thus, our results may not be generalisable to caregivers of those with other EDs, who may face different challenges and support needs [21, 32]. Further studies should include a more diverse group of participants, encompassing all forms of EDs. A multi-centre design would probably address this issue and then enhance the generalisability of the findings. Third, caregivers enrolled in the programme spontaneously. Approximately 10% of caregivers of patients treated at the recruitment centre benefited from the intervention. This may have introduced a selection bias. It may be hypothesised that caregivers who participated were possibly those experiencing higher distress. Additionally, the positive outcomes observed in this group, potentially facing severe challenges, are encouraging. However, further research is needed to evaluate the effects of systematically offering the programme to all caregivers at the inclusion centres and to determine whether similar benefits extend to those facing less severe challenges.

Finally, it is crucial to consider that the participants may have found in the BREF-ED programme a space for tension release, access to information about the EDs, and thus relief from feelings of hopelessness in an acute context. This supportive function of the intervention has likely contributed to the improvement in self-reported burden observed. It is essential to consider that these short-term improvements may be dependent on the acute context, and they may not necessarily lead to lasting benefits [30, 76]. Delayed effects of the intervention may also occur [10, 77]. Therefore, medium- and long-term follow-ups are needed to draw conclusions about the lasting effect of the BREF-ED programme.

### Future research

Future developments of the programme should include strengthening collaboration with local peer-support and family organisations that offer long-term assistance and resources to informal caregivers of individuals with EDs, as well as with healthcare professionals who facilitate long-format group FPE programmes. This could further alleviate caregiver burden, enhance the programme’s effectiveness, and sustain its clinical benefits. Indeed, existing literature on caregiver interventions highlights the value of peer group activities in promoting the exchange of experiences and coping strategies among caregivers, as well as providing emotional support [10, 11, 34]. Similarly, long FPE programmes have demonstrated their effectiveness in long-term improvement of caregivers’ psychological distress in the context of EDs [77]. Furthermore, to facilitate the broader dissemination of the BREF-ED programme, future studies should explore the feasibility of a remote and digital-based version. A significant number of caregivers do not have access to healthcare centres [48] and face challenges in attending in-person sessions. Technology could bridge these gaps by offering greater flexibility and access to early FPE support [49, 78–82], such as the BREF-ED programme.

## Conclusion

The BREF-ED programme for caregivers demonstrated high adherence rates, strong satisfaction, and positive feedback regarding its utility, supporting its feasibility for effective implementation. Preliminary findings also suggest benefits in reducing caregivers’ burden and related depressive symptoms in acute contexts. Given its time and resource efficiency, the programme appears easy to disseminate and may contribute to the development of a structured support pathway for caregivers.

## Supporting information

10.1192/j.eurpsy.2025.10089.sm001 Scanferla et al. supplementary material Scanferla et al. supplementary material

## Data Availability

The datasets analysed in this study and which support its conclusions are available from the corresponding author upon reasonable request.

## Supplementary material

The supplementary material for this article can be found at http://doi.org/10.1192/j.eurpsy.2025.10089.
