# Supplementary material for: Short-term impact of BREF-ED, an early, single-family psychoeducational programme for caregivers of individuals with eating disorders: A retrospective pilot study
Source: Eur Psychiatry. 2025 Aug 22;68(1):e121. doi: 10.1192/j.eurpsy.2025.10089 (PMC12438994; doi:10.1192/j.eurpsy.2025.10089)
Supplement: Scanferla et al. supplementary material [file S0924933825100898sup001.docx]

**Short-term impact of BREF-ED, an early, single-family psychoeducational programme for caregivers of individuals with eating disorders: a retrospective pilot study**

**Supplementary analysis**

**Table S1**. Pre vs post: 2x2 contingency table (CES-D ≥ 16).

|  |  | Post | |  |
| --- | --- | --- | --- | --- |
|  |  | Depressed | Not depressed | Total |
| Pre | Depressed | 12 (23.1%) | 22 (42.3%) | 34 (65.4%) |
|  | Not depressed | 3 (5.8%) | 15 (28.8%) |  |
|  | Total | 15 (28.8%) |  |  |
|  |  |  |  |  |
|  |  | Follow-up | |  |
|  |  | Depressed | Not depressed | Total |
| Pre | Depressed | 8 (15.4%) | 26 (50%) | 34 (65.4%) |
|  | Not depressed | 5 (9.6%) | 13 (25%) |  |
|  | Total | 13 (25%) |  |  |
|  |  |  |  |  |
|  |  | Follow-up | |  |
|  |  | Depressed | Not depressed | Total |
| Post | Depressed | 8 (15.4%) | 7 (13.5%) | 15 (28.8%) |
|  | Not depressed | 5 (9.6%) | 32 (61.5%) |  |
|  | Total | 13 (25%) |  |  |

**Table S2**. Pre vs post: 2x2 contingency table (ZBI ≥ 41).

|  |  | Post | |  |
| --- | --- | --- | --- | --- |
|  |  | Burdened | Not burdened | Total |
| Pre | Burdened | 6 (11.5%) | 11 (21.2%) | 17 (32.7%) |
|  | Not burdened | 1 (1.9%) | 34 (65.4%) |  |
|  | Total | 7 (13.5%) |  |  |
|  |  |  |  |  |
|  |  | Follow-up | |  |
|  |  | Burdened | Not burdened | Total |
| Pre | Burdened | 7 (13.5%) | 10 (19.2%) | 17 (32.7%) |
|  | Not burdened | 1 (1.9%) | 34 (65.4%) |  |
|  | Total | 8 (15.4%) |  |  |
|  |  |  |  |  |
|  |  | Follow-up | |  |
|  |  | Burdened | Not burdened | Total |
| Post | Burdened | 5 (9.6%) | 2 (3.8%) | 7 (13.5%) |
|  | Not burdened | 3 (5.8%) | 42 (80.8%) |  |
|  | Total | 8 (15.4%) |  |  |
